# *An aco-2::gfp* knock-in enables the monitoring of mitochondrial morphology throughout *C. elegans* lifespan.

**DOI:** 10.17912/micropub.biology.000599

**Published:** 2022-07-17

**Authors:** David V. Begelman, Georgia Woods, Dipa Bhaumik, Suzanne Angeli, Anna C Foulger, Mark Lucanic, Jianfeng Lan, Julie K. Andersen, Gordon J. Lithgow

**Affiliations:** 1 The Buck Institute for Research on Aging; 2 University of Maine, Molecular & Biomedical Sciences; 3 GeroStateAlpha; 4 Guangxi Key Laboratory of Molecular Medicine in Liver Injury and Repair, the Affiliated Hospital of Guilin Medical University, Guilin, 541001, Guangxi, China

## Abstract

We used CRISPR/Cas9 gene editing in *C. elegans* in order to fluorescently tag endogenous aconitase-2 (ACO-2). ACO-2 is a mitochondrially localized protein, and the *aco-2::gfp* strain enabled the examination of native mitochondrial morphology in live animals. Here we validate that the *aco-2::gfp* strain displays the prototypic changes in mitochondrial morphology known to occur during aging and upon paraquat (PQ) induced mitochondrial stress. We also provide evidence that the ACO-2::GFP reporter can serve as a superior means for tracking mitochondrial morphology than conventional MitoTracker dyes—especially in aged-worms.

**Figure 1. Characterization of aco-2::gfp C. elegans strain:**
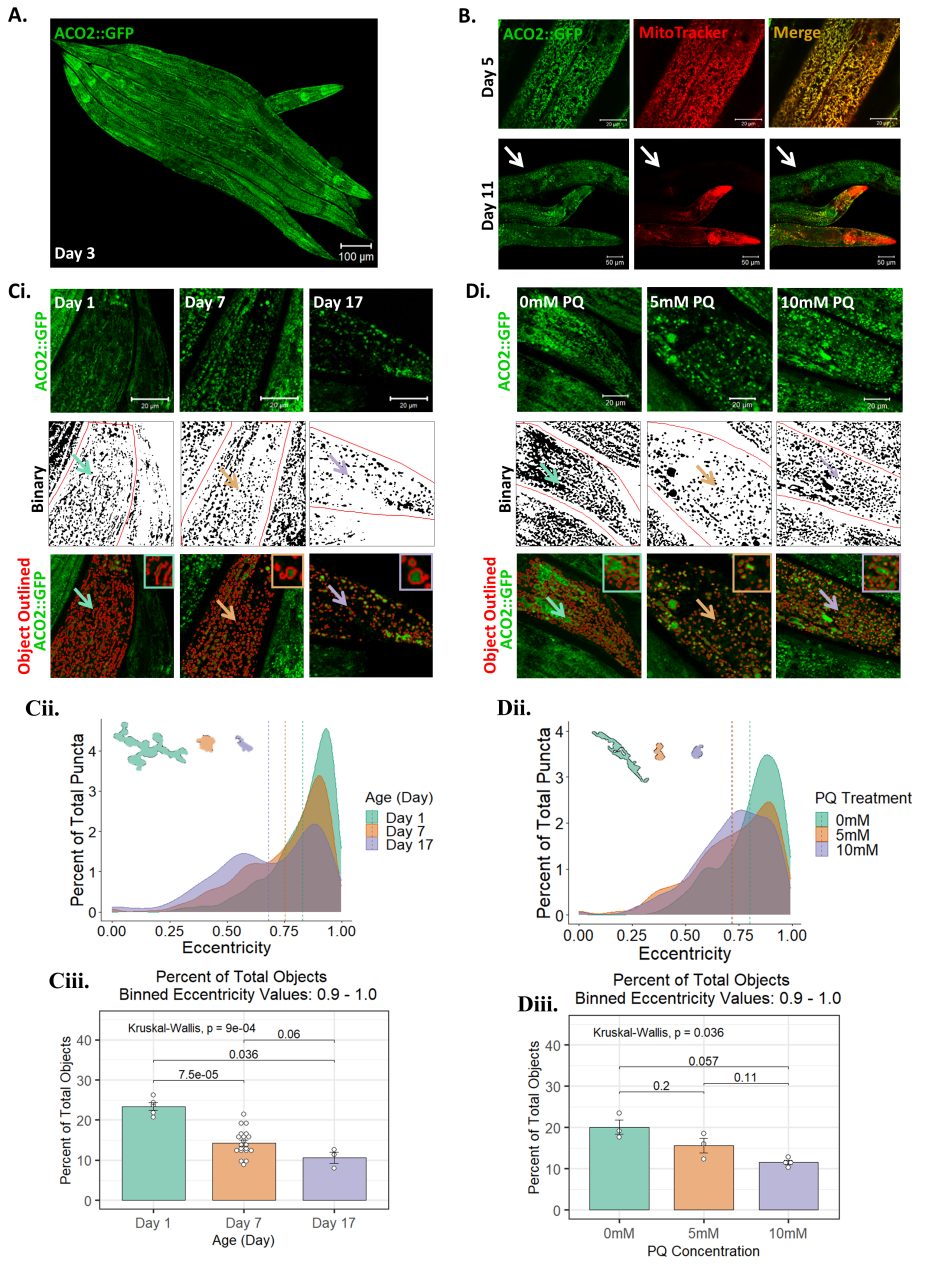
**(A)** Confocal imaging of five (aligned) Day 3 adults, showing ACO-2::GFP fluorescence throughout the worms and their developing germline (scale bar 100 µm). **(B)** ACO-2::GFP (green) and MitoTracker Red (red) staining in Day 5 (top panels) and Day 11 (bottom panels) worms. In Day 5 worms, colocalization (yellow) is pronounced, but by Day 11 there are large portions of the worm devoid of MitoTracker Red staining (arrow). Top panel scale bar 20 µm and bottom panel scale bar 50 µm. **(C)** Fragmentation and rounding of the mitochondrial network upon aging. **(i.)** Confocal images (max projections) of the tail region of *aco-2::gfp* worms (green top panels) across representative ages: Day 1 (left panels); Day 7 (middle panels); and Day 17 (right panels). Binary images help visualize the mitochondrial network (black and white middle panels), and individual mitochondrial ‘objects’ were identified using an automated image analysis pipeline (red outlines, bottom panels), and image insets in the top right corner show a zoom-in of corresponding ‘example objects’ indicated with colored arrows (Scale bar = 20 µm.) **(ii.)** A density-plot of object *eccentricity* across age *.* For each mitochondrial object ‘ *eccentricity* ’ was measured, where *eccentricity* values approaching 1 correspond to shapes which are more 'elongated' (vs. more 'rounded'—where a perfectly circular object has a value of 0). Dashed vertical lines identify the mean value for object eccentricity at Day 1 (green line); Day 7 (orange line); and Day 17 (purple line). Example objects from Day 1 (green object), Day 7 (orange object), and Day 17 (purple object) are displayed on the upper left side of the graph. **(iii.)** Bar graph of the percent of ‘elongated objects’ (those with an eccentricity value between 0.9-1.0). This demonstrates that there is a significant and progressive drop in the proportion of mitochondria that have an elongated/tubular morphology from Day 1 (green bar) to Day 7 (orange bar) to Day 11 (purple bar), consistent with the mitochondrial network becoming more fragmented and rounded as the worms age. Data is plotted as the mean ± SEM, each point is a worm, n = 3 - 20 worms, *p* values calculated using Kruskal-Wallis test, *p* = 0.0009. **(D)** Fragmentation and rounding of the mitochondrial network upon paraquat (PQ) exposure. **(i.)** Confocal images (max projections) of the tail region of *aco-2::gfp* worms (green top panels) after exposure to PQ for 48 hours starting at Day 1: 0 mM (left panels); 5 mM (middle panels); and 10 mM (right panels). As in panel ‘C’, binary images are shown (middle panels), as are identified mitochondrial ‘objects’ (bottom panels). (Scale bar = 20 µm.) **(ii.)** A density plot of object *eccentricity* upon PQ exposure *.* For each mitochondrial object ‘ *eccentricity* ’ was measured as described in panel ‘C’. Dashed vertical lines identify the mean value for object eccentricity with 0 mM (green line); 5 mM (orange line); and 10 mM (purple line). Example objects from 0 mM (green object), 5 mM (orange object), and 10 mM (purple object) are displayed on the upper left side of the graph. **(iii.)** Bar graph showing the percent of ‘elongated objects’, demonstrating that PQ causes a significant reduction in the proportion of mitochondria that have an elongated/tubular morphology: 0 mM (green bar); 5 mM (orange bar); 10 mM (purple bar). Data is plotted as the mean ± SEM, each point is a worm, n = 3 - 4 worms, p values calculated using Kruskal-Wallis test, p = 0.036.

## Description

Mitochondrial morphology and dynamics invariably change as cells age (Regmi *et al.* 2014; Iqbal *et al.* 2013; Bua *et al.* 2002; Beregi *et al.* 1988). Studies have shown that in the context of aging, *C. elegans* mitochondria undergo fragmentation and loss of volume (Gaffney *et al.* 2018; Kim *et al.* 2018; Regmi *et al* . 2014; Johnson and Nehrke 2010; Yasuda *et al.* 2006). Therefore, the preservation of ‘youthful-looking’ mitochondria is routinely interpreted as a sign of healthy aging. To develop a convenient and reliable *in vivo* model to examine native mitochondrial morphology throughout the lifespan of the worm, we targeted ACO-2 *,* a protein encoded in the nuclear genome *.* ACO-2 is an aconitase, with hydratase activity, which localizes to the mitochondrial matrix *of C. elegans* where it functions in the TCA cycle. ACO-2 is expressed at relatively high-levels and is functional as a monomeric species (Slaughter, Hopkinson, and Harris 1975; 1977). In order to generate a C-terminally tagged ACO-2 reporter strain, we used CRISPR/Cas9 genome editing and inserted *gfp* at the 3’ end of *aco-2* locus.

As predicted, confocal imaging revealed that ACO-2::GFP is easily detected, as it is highly and ubiquitously expressed throughout *C. elegans* adult tissues and in the developing germline **(Figure 1A)** . The *aco-2::gfp* worms looked normal and healthy throughout their lifespan and displayed no overtly altered phenotypes. In Day 1 adults, the reporter reliably labeled mitochondria, evidenced by the fact that GFP fluorescence and MitoTracker Red staining were completely colocalized **(Figure 1B—Day 1)** . However, by Day 11, ACO-2::GFP continued to label putative mitochondria, however MitoTracker Red staining had drastically decreased, and was no longer acting as an efficient mitochondrial marker **(Figure 1B—Day 11)** . The reason dyes such as MitoTracker do not efficiently stain mitochondria in aged worms is twofold: (1) mitochondrial staining depends upon ingestion of the dye during ‘pharyngeal pumping’— *and* pumping rates drop precipitously in aged-animals (Huang *et al.* 2004); and (2) mitochondrial staining depends upon the organelle’s ability to maintain a negative membrane potential— *and* aged-mitochondria lose this capacity, becoming relatively depolarized (Gaffney *et al.* 2018). Therefore, compared to MitoTracker dyes, the *aco-2::gfp* reporter strain is a more reliable means to monitor mitochondrial morphology in aged-animals.

To assess whether our reporter strain faithfully exemplifies the known age-related changes in mitochondrial morphology (namely network fragmentation and rounding) we developed an automated imaging pipeline (using *CellProfiler* and *R* ) for quantifying mitochondrial eccentricity in the *aco-2::gfp* model. (Many similar automated programs exist for assessing mitochondrial morphology, including a recently developed program, “MitoSegNet” which is a ‘deep learning’ image analysis tool, capable of identifying individual mitochondrial segments with high accuracy (Fischer *et al.* 2020).) Using our own program, we assessed morphology at Day 1, Day 7, and Day 17. The mitochondria of Day 7 and Day 17 animals significantly shifted towards a circular, punctate morphology compared to the more tubular, ellipsoid morphology of the Day 1 adults **(Figure 1C)** .

Beyond age-related changes in mitochondrial morphology, changes stemming from cellular stress are also routinely assessed and of general interest. For example, the environmental toxin, paraquat (PQ), is a free radical generator that causes oxidative stress resulting in compromised mitochondrial function (Bus and Gibson 1984) and in *C. elegans* PQ pushes mitochondria from a more linear to a more fragmented-circular morphology, reminiscent of the changes that occur with aging (Bora *et al.* 2021). As with aging, many mitochondrial toxins including PQ induce depolarization of mitochondria, which precludes the use of membrane-potential dependent dyes to assess morphology (Diez *et al.* 2021). To assess morphometric changes in the *aco-2::gfp* strain upon PQ-mediated stress, we subjected Day 1 *aco-2::gfp* worms to PQ for 48 hours. We observed a dramatic increase in the quantity of punctate or circular objects and a decrease in tubular, interconnected filamentous objects within the 5 mM and 10 mM PQ treatment groups **(Figure 1D)** . These data demonstrate that our model serves as a useful tool for measuring age-related and stress-mediated changes in mitochondrial morphology.

We have developed an ACO-2::GFP reporter and established that it enables visualization of the prototypic changes in mitochondrial morphology known to occur with aging and cellular stress. As demonstrated, the ACO-2::GFP reporter can serve as a superior means for tracking mitochondrial morphology than conventional ‘mito-dyes’—especially in aged-animals. Importantly, membrane-potential dependent dyes (such as MitoTracker and TMRM) can indicate the functional status of the mitochondria, and conversely, ACO-2::GFP positive organelles may not always identify healthy, polarized and fully functional mitochondria, particularly in aged-animals. Though to reiterate, in aged *C. elegans* it is problematic to infer that a mitochondria is non-functional because it is not staining with a membrane-potential dependent dye, as lack of staining could be trivially explained by failure of old worms to ingest the dye.

Additionally, while many fluorescent mitochondrial reporter strains (e.g. GFP ^mt^ ) exist in worms, commonly these are multi-copy-tandem-repeat transgenic strains, resulting in pronounced overexpression of the transgene. In order for these fusion proteins to maintain their normal function and fluoresce, they must be correctly folded within the mitochondria with the aid of resident chaperones; this places a high burden on mitochondrial machinery and can lead to an increase in mitochondrial biogenesis and altered mitochondrial unfolded protein response (UPR ^mt^ ) dynamics (Shpilka *et al* . 2021). As such, using many of the existing reporter strains to monitor native mitochondrial morphology is potentially problematic. Instead, a single copy fluorescent-fusion-knock-in, as we have generated here, is likely to more faithfully recapitulate native mitochondrial morphology (Fire *et al.* 1998). Furthermore, *aco-2::gfp* is also an ideal reporter strain because ACO-2 is endogenously expressed at high levels, and consequently ACO-2::GFP fluorescence is easily detected.

To clarify, we have not directly tested the functionality of ACO-2::GFP in this study, however we would like to highlight circumstantial evidence that supports the idea that, *if* ACO-2::GFP function is altered in some way, those alterations are likely to have only subtle impacts on ACO-2 function and, consequently, mitochondrial morphology. As described earlier, *aco-2::gfp* animals generally look normal and healthy and, in young animals where the experiment is feasible, ACO-2::GFP and MitoTracker Red are completely colocalized. This suggests that fusing GFP to the C-terminus of ACO-2 has no major impact on ACO-2’s folding and/or targeting to the mitochondria. Furthermore, our own unpublished data demonstrate that RNAi mediated knock-down of *aco-2* causes a significant lifespan extension (a ~+20% in median lifespan relative to N2); additional preliminary data from our lab indicate that the *aco-2::gfp* strain has a normal lifespan compared to N2 animals. Taken together these data suggest that ACO-2::GFP function is not severely compromised. In this study we have *not* tested whether the fusion of GFP to ACO-2 disrupts mitochondrial morphology and dynamics, however we have shown that in the context of aging and upon mitochondrial stress, the mitochondrial network in *aco-2::gfp* worms is behaving as would be predicted.

In conclusion, the *aco-2::gfp* *C. elegans* strain, characterized here, represents a complementary tool for drug discovery that may be used to expedite the development of therapeutics that protect against mitochondrial dysfunction and mitigate age-related pathologies.

## Methods

***Generation of CRISPR-mediated-aco-2::gfp knock-in*** : Strain GL390, Genotype: aco-2( *rf40* ). *rf40* is *aco-2::gfp* , CRISPR knock-in of GFP at the C-terminus. The homology arm repair template and guide RNAs were designed such that *gfp* would be inserted right before the STOP codon of *aco-2* . There are two *aco-2* isoforms predicted, however they differ in their N-terminal regions, and share the same STOP, so our knock-in is predicted to tag both isoforms, generating two C-terminally tagged ACO-2::GFP variants. We used the following program (http://crispr.mit.edu/) to design two distinct sgRNAs (sgRNA1: **ATCTGCCCTCAACAGAATGA** and sgRNA2: **GCAACAAATCGAGTGGTTCA** ). We injected 25 N2 worms with the following plasmid cocktails: (1) *aco-2 targeted* sgRNA (50 ng/µL); (2) homology arm repair template (50 ng/µL) and injection markers (3) *rol-6* (50 ng/µL) and (4) *myo-2p::mCherry* (5ng/µL). Using a compound fluorescent microscope, we selected 4 independently injected lines that continued to fluoresce green at the F3 generation. One of these 4 lines was chosen at random and backcrossed 6X into N2, to generate the final strain that was used in subsequent experiments. Insertion of *gfp* into the *aco-2* locus was confirmed by PCR using primers: 5’- ACAAGGAATGCTCCCACTCACAT-3’ and 5’- CTTACCCATGGAACAGGTAGT-3’.

***MitoTracker Red:*** Worms were soaked in 50 µL MitoTracker Red CMXRos (2.5 µM in M9 buffer; Invitrogen M7512) for 10 minutes at 20°C in the dark. Worms were then transferred to an OP50-seeded NGM plate and allowed to recover for 2 hours at 20°C in the dark.

***Paraquat treatment:*** Day 1 adult worms were treated with 0, 5 or 10 mM PQ (dissolved in water) for 48 hours at 20°C before imaging.

***Confocal microscopy:*** Worms were anesthetized with 5 mM levamisole and mounted on 2 % agarose pads on glass slides. All images were acquired using a Zeiss LSM 780 inverted confocal microscope. Tile images of PQ treated worms were taken using Plan-Neofluar 20X/0.50 Ph2 objective at 1X digital zoom. Using EC Plan-Neofluar 40X/1.30 Oil Ph3 objective, a z-stack spanning *C. elegans* tails were acquired using 256 by 256 pixel – 8 bit image at 1X digital zoom with 1 µm steps. For the aging paradigm data, tile images were taken using the Plan-Apochromat 63X/1.40 Oil DIC objective at 0.6X digital zoom. Z-stack images spanning *C. elegans* tails were completed using the Plan-Apochromat 63X/1.40 Oil DIC objective at 512 by 512 pixel – 8 bit image at 2X digital zoom with 2 µm steps. A 488 nm laser was used to visualize GFP signal. Using the Zeiss ZEN software, max projection images were created and GFP expression brightness contrast was enhanced. The same parameters were used for all images.

***Morphometric image analysis:*** Max projection images were analyzed using CellProfiler, an automated image analysis software (Version 4.2.1; Broad Institute). Briefly, imported images were converted to grayscale using the “ColorToGray” module. Using the “IdentifyObjectsManually” module, the tail regions of *C. elegans* was manually selected and masked via “MaskImage” module. All GFP objects within the annotated tail region were selected using the “IdentifyPrimaryObjects” module with the following settings:

- Typical diameter of objects in pixel units (Min, Max): 30 – 500
- Objects outside of diameter range are discarded.
- Objects touching the border of the image are not discarded.
- Threshold strategy and method: Adaptive, Otsu (Two-Class)
- Threshold smoothing scale, correction factor, adaptive window: 1.3488, 1, 10
- Lower and upper bounds on threshold: 0 – 1
- Log transformation after thresholding was not utilized.
- Clumped objects were distinguished based on intensity.
- Dividing lines between clumped objects were drawn based on intensity.
- Smoothing filer of 25 pixels was used.
- Minimum allowed distance between local maximum was automatically calculated.
- After thresholding and declumping, holes in identified objects were filled.
- Lower resolution images were not used to speed up the automated quantification procedure.
- Local maxima were not displayed.

After object identification, the following parameter was recorded using the “MeasureObjectSizeShape” module:

- Eccentricity: Calculated as the distance between the ellipse's foci and its major axis length. The eccentricity value of 1 indicates a tubular, ellipsoid object, whereas 0 indicates circular puncta.

The recorded parameters were graphed using R (Version 4.1.1). The R plots were created using ggplot2 and ggpubr packages. For the statistical analysis, a Kruskal–Wallis one-way analysis of variance (ANOVA) with multiple comparisons was used to assess for significance among the groups.

***Author Contributions*** :

David V. Begelman: Wrote and edited manuscript. Conducted confocal imaging on aged and PQ treated worms and developed and conducted all automated image analysis and generated all graphs.

Georgia Woods: Wrote and edited manuscript. Conducted confocal imaging and helped with analysis.

Suzanne Angeli: Conducted MitoTracker experiments.

Dipa Bhaumik: Edited manuscript. Engineered the *aco-2::gfp* strain and helped design and cary out experiments.

Anna C. Foulger: Prepared, handled and back crossed the *aco-2::gfp* strain.

Mark Lucanic: Helped with engineering the *aco-2::gfp* strain.

Jianfeng Lan: Helped with engineering the strain.

Julie K. Andersen: Participated in overseeing planning of experiments and edited manuscript.

Gordon J. Lithgow: Participated in overseeing planning of experiments and edited manuscript.
